# Acute Laryngeal Dystonia Mimicking Anaphylaxis Following Antipsychotic Initiation: A Case Report

**DOI:** 10.1155/crps/9856544

**Published:** 2026-07-11

**Authors:** Devina Wadhwa

**Affiliations:** ^1^ Department of Psychiatry, Thunder Bay Regional Health Sciences Centre, Thunder Bay, Ontario, Canada, tbrhsc.net

**Keywords:** acute laryngeal dystonia, anaphylaxis, antipsychotic adverse effect, case report, lurasidone

## Abstract

Acute laryngeal dystonia (ALD) is an uncommon but potentially life‐threatening adverse effect of dopamine D2 receptor–blocking agents. Because airway symptoms predominate, it may be misdiagnosed as anaphylaxis. A 24‐year‐old woman developed recurrent episodes of throat tightness, dysphonia and stridor within days of antipsychotic initiation and subsequent dose escalation. Initial episodes were treated as anaphylaxis based on airway symptoms and mild tachycardia. Serum tryptase was mildly elevated, further contributing to diagnostic uncertainty. However, the absence of cutaneous findings or hypotension, recurrence after dose escalation and temporal association with antipsychotic therapy prompted reconsideration of the diagnosis. Rapid resolution following intramuscular benztropine supported the diagnosis of ALD as the most likely diagnosis. This case highlights the importance of medication history, temporal pattern recognition and therapeutic response in distinguishing dystonia from allergic reactions. It also illustrates how anchoring bias and diagnostic momentum may contribute to recurrent misclassification. Early recognition of laryngeal dystonia can prevent unnecessary treatment, reduce the risk of airway compromise, and improve diagnostic accuracy.

**Summary**

• Acute laryngeal dystonia (ALD) can present with stridor and airway distress within days of antipsychotic initiation or dose escalation.
• The absence of urticaria, angioedema, and hypotension should prompt reconsideration of anaphylaxis.
• Rapid resolution after anticholinergic therapy supports the diagnosis of dystonia.
• Recurrent presentations warrant diagnostic reappraisal to mitigate anchoring bias and diagnostic momentum.

## 1. Introduction

Acute dystonic reactions are well‐recognized adverse effects of dopamine D2 receptor‐blocking agents [1–3]. Acute laryngeal dystonia (ALD) represents a rare but potentially fatal manifestation involving involuntary contraction of the laryngeal musculature, resulting in stridor, dysphonia, dysphagia, and respiratory distress [2, 3]. Because airway symptoms predominate, ALD may be mistaken for allergic, respiratory, or anxiety‐related conditions [3].

A recent systematic review identified ALD as an uncommon but important adverse effect associated with both first‐ and second‐generation antipsychotics, with delayed recognition frequently reported because of its resemblance to other causes of airway compromise [3]. A case of recurrent ALD is described initially diagnosed as anaphylaxis, highlighting the role of diagnostic reasoning and cognitive bias in clinical decision‐making.

## 2. Case Presentation

A 24‐year‐old woman with a prior psychiatric history of depression was admitted for a first episode of mania occurring several months after the treatment of major depressive disorder with sertraline. The antidepressant was discontinued and lurasidone 20 mg daily was initiated as a treatment choice for Bipolar illness, the patient was presented with various options for bipolar illness treatment based on the Canadian Bipolar Guidelines, however, wanted a trial of lurasidone given its side effect profile and patient preference [4].

Within 48 h of initiation, she developed acute throat tightness, dysphonia, subjective choking, and inspiratory stridor. Oxygen saturation remained within the normal range throughout the episode. She denied rash, urticaria, facial swelling, wheezing, or gastrointestinal symptoms. Blood pressure was 120/85 mmHg, and heart rate was 105 beats/min.

Given concern for anaphylaxis to a presumed food allergen, she was transferred to the intensive care unit for an observation period of 6 and treated with epinephrine and diphenhydramine, with subsequent improvement. Serum tryptase obtained following the episode was mildly elevated at 15 ng/mL (reference range <11.4 ng/mL). The exact timing of sample collection relative to symptom onset was unavailable for review. She had no known drug or food allergies.

Lurasidone was continued. Two weeks later, the dose was increased to 40 mg daily. Seven days after dose escalation, she presented to the emergency department with a recurrence of identical symptoms. Vital signs again showed normal blood pressure with mild tachycardia. She received epinephrine and diphenhydramine and was discharged with a presumptive diagnosis of recurrent anaphylaxis.

The following day, she returned with persistent symptoms. Given the temporal association with recent antipsychotic initiation and dose escalation, and the absence of cutaneous manifestations or hypotension during prior episodes, an acute dystonic reaction was considered. She was administered intramuscular benztropine 2 mg. Within 5 min, her throat tightness and dysphonia were resolved completely.

No additional extrapyramidal symptoms, including cervical dystonia, tongue protrusion, jaw spasm, or oculogyric crisis, were observed during any of the episodes. Lurasidone was discontinued, and a short course of oral benztropine was prescribed. No further episodes occurred. The final diagnosis was ALD secondary to antipsychotic therapy. Lithium was subsequently initiated as an alternative treatment and was tolerated well. The patient’s clinical course is summarized in Table 1.

**Table 1:** Timeline of events.

Time	Clinical event
Day 0	Lurasidone 20 mg initiated
Day 2	First episode of throat tightness, dysphonia, and stridor
Day 2	Treated as presumed anaphylaxis with epinephrine and diphenhydramine
Week 2	Lurasidone increased to 40 mg daily
Week 3	Recurrence of identical symptoms
Week 3	Treated again as presumed anaphylaxis
Following day	Reassessment and consideration of acute dystonia
Following day	Benztropine 2 mg IM administered
Within 5 min	Complete symptom resolution
After discontinuation	No recurrence; lithium initiated

## 3. Discussion

Acute dystonic reactions are well‐recognized adverse effects of dopamine D2 receptor‐blocking agents and most commonly occur within 72 h of treatment initiation or dose escalation [1, 2]. Younger age is a known risk factor [2]. Although cervical dystonia and oculogyric crisis are more frequently reported, involvement of laryngeal musculature is uncommon but potentially life‐threatening because of airway compromise [2, 3].

ALD results from a dopaminergic blockade in the nigrostriatal pathway, producing relative cholinergic excess and sustained involuntary muscle contraction [1, 2]. When pharyngeal or laryngeal muscles are affected, patients may present with stridor, dysphonia, dysphagia, and subjective air hunger [2, 3]. Because airway symptoms predominate, the presentation may be mistaken for allergic or respiratory pathology [3].

A recent systematic review of antipsychotic‐associated ALD highlighted the rarity of this adverse effect and emphasized frequent diagnostic delays due to clinical overlap with allergic, respiratory, and anxiety‐related conditions [3]. Our case contributes to this literature by demonstrating repeated misclassification of anaphylaxis despite recurring medication‐associated symptoms.

The initial working diagnosis of anaphylaxis was clinically plausible. Airway symptoms and mild tachycardia appropriately prompted concern for a potentially life‐threatening allergic reaction. Furthermore, serum tryptase was mildly elevated at 15 ng/mL. However, the significance of this finding remains uncertain because the timing of sample collection relative to symptom onset was unavailable. Although elevated tryptase may support mast‐cell activation, interpretation requires clinical context [5–7]. In this case, the absence of urticaria, angioedema, hypotension, gastrointestinal symptoms, or a clear allergen exposure reduced the likelihood of anaphylaxis [5, 6].

The interpretation of the initial treatment response also warrants consideration. Improvement followed administration of both epinephrine and diphenhydramine. While initially viewed as supporting an allergic etiology, diphenhydramine possesses clinically significant anticholinergic properties and is commonly used in the treatment of acute dystonic reactions [1, 2]. Consequently, improvement cannot be attributed solely to epinephrine and may have reflected partial treatment of an evolving dystonic process.

Lurasidone possesses an elimination half‐life of ~18–37 h and reaches steady‐state concentrations within approximately 7 days [8]. The recurrence of symptoms 1 week following dose escalation is therefore consistent with increasing drug exposure and the established temporal pattern of acute dystonic reactions after antipsychotic initiation or dosage increase.

The response to anticholinergic therapy is both therapeutic and diagnostically informative in suspected dystonia. Intramuscular or intravenous anticholinergic agents, including diphenhydramine and benztropine (1–2 mg), typically produce rapid improvement within minutes [1, 2]. In this patient, complete resolution within 5 min strongly supported the diagnosis of an acute dystonic reaction.

This case also illustrates the influence of cognitive bias in diagnostic decision‐making. Anchoring bias occurs when clinicians remain fixed on an initial diagnostic impression despite contradictory information [9]. Diagnostic momentum refers to the persistence of a diagnostic label across successive clinical encounters [9, 10]. Once anaphylaxis had been assigned, subsequent presentations were interpreted through the same framework despite atypical features and recurrent association with medication exposure.

Early recognition of laryngeal dystonia is essential because untreated cases may progress to complete airway obstruction [3]. Conversely, repeated administration of epinephrine may expose patients to unnecessary cardiovascular risk and reinforce an incorrect diagnostic label. Careful attention to medication history, temporal relationships, and therapeutic response can assist clinicians in distinguishing dystonia from allergic reactions and prevent recurrent misdiagnosis. The distinguishing clinical features of ALD and anaphylaxis are summarized in Table 2.

**Table 2:** Differentiating laryngeal dystonia from anaphylaxis.

Feature	Laryngeal dystonia	Anaphylaxis
Timing	24–72 h after antipsychotic initiation or dose increase	Minutes after allergen exposure
Cutaneous findings	Absent	Common
Blood pressure	Typically normal	Often reduced
Tryptase	Normal or mildly elevated	Often elevated (1–4 h window)
Response to anticholinergic	Rapid resolution	No response
Response to epinephrine	Variable or limited	Marked improvement

ALD is substantially less common than anaphylaxis and may be unfamiliar to many clinicians [3]. This disparity in familiarity may itself contribute to diagnostic error, as rare diagnoses are more easily overlooked when more common explanations appear initially plausible.

This case has several limitations. Otolaryngology consultation and laryngoscopic examination were not performed, preventing direct visualization of laryngeal dysfunction. Additionally, the timing of serum tryptase measurements relative to symptom onset was unavailable, limiting the interpretation of this laboratory finding. Despite these limitations, the temporal relationship to lurasidone initiation and dose escalation, recurrence of stereotyped symptoms, absence of typical anaphylactic features, rapid resolution following benztropine administration, and complete symptom remission following lurasidone discontinuation support ALD as the most likely diagnosis.

## 4. Conclusion

ALD is a rare but potentially life‐threatening adverse effect of antipsychotic treatment that may mimic anaphylaxis. Careful attention to medication history, temporal relationships, and response to anticholinergic therapy can facilitate prompt recognition and prevent recurrent misdiagnosis and unnecessary interventions.

## Funding

No funding was received for the preparation of this manuscript.

## Disclosure

This case report was prepared in accordance with the CARE guidelines. A completed CARE checklist accompanies this submission.

## Ethics Statement

Ethical approval was not required for this case report in accordance with local institutional policies. All identifying information has been removed to protect patient confidentiality.

## Consent

Written informed consent was obtained from the patient for the publication of this case report and any accompanying data.

## Conflicts of Interest

The author declares no conflicts of interest.

## Data Availability

Research data are not shared.
